# Ultrasound Study of Trocar Site Hernia after Bariatric Surgery: Medium and Long-Term Follow-Up

**DOI:** 10.3389/jaws.2025.15002

**Published:** 2025-07-28

**Authors:** Emilio López-Negrete Cueto, Aida Suárez Sánchez, Jose Luis Rodicio Miravalles, Sonia Amoza Pais, María Moreno Gijón, Sandra Sanz Navarro, Tamara Díaz Vico, Pablo Noriega Menéndez, Ana Montes García, Lourdes Sanz Álvarez

**Affiliations:** ^1^Division of General Surgery, Hospital Universitario Central de Asturias (HUCA), Oviedo, Spain; ^2^Division of General Surgery, Hospital Universitario de Cabueñes (HUCAB), Gijón, Spain; ^3^Radiology Department, Hospital Universitario Central de Asturias (HUCA), Oviedo, Spain

**Keywords:** laparoscopy, trocar site hernia, bariatric surgery, ultrasound, bladeless trocar

## Abstract

**Introduction:**

Patients undergoing bariatric surgery have comorbidities and risk factors which favour incisional hernias. Bladeless trocars are safe and not very harmful, being the preferred choice without port closure. The objective of this study is to evaluate the true occurrence of trocar hernias (trocar site hernia) in these patients in medium and long term.

**Material and Methods:**

We made an observational, descriptive, longitudinal, and retrospective study in patients who underwent bariatric surgery by laparoscopy with 12 mm bladeless trocars without fascial closure. We selected patients who agreed to participate in the study between January 2015 and July 2016. We also collected data on risk factors, pain scale, and ultrasound of port-site by an expert radiologist in 2018 and 2022.

**Results:**

In the first period 45 patients were included, with a mean age of 49.9 years and an initial BMI of 45.5 kg/m^2^. Most of them (88.9%) were operated by gastric bypass. Forty-five abdominal wall ultrasounds were performed in 2018 with findings of 7 trocar site hernia for a total of 185 explored ports (3.8%), all of them in the epigastric port (p < 0.001) and asymptomatic. In 2022, 32 ultrasound detected 7 trocar site hernia (4.4%), 3 new and 4 knowns, with 2 symptomatic patients at the trocar site hernia with mild symptoms not consulted.

**Conclusion:**

The incidence of trocar hernia in the medium and long term in postoperative bariatric surgery with 12 mm bladeless trocars without fascial closure is low, all of them being in the midline and paucity of symptoms. Our primary approach involves abstaining from closing the transmuscular accesses, deeming it unnecessary, while evaluating the closure or lateralization of the midline trocar.

## Introduction

As is well known, bariatric surgery has grown globally due to increasing obesity prevalence [1]. Given the insufficiency of preventive measures, bariatric surgery has been shown to be the most effective long-term solution for tackling this problem [2, 3]. The most commonly performed techniques worldwide are sleeve gastrectomy and gastric bypass, with the latter being the most frequently utilized at our hospital [4], experiencing a gradual escalation in the volume of procedures performed due to the worldwide increase in obesity rates [5].

Incisional hernias represent one of the potential complications of these procedures; however, there exists considerable variability in the reported incidence of such hernias across published articles to date, ranging from 0.9% to 68.8% according to different publications due to being patients at high risk of developing hernias [6–10].

A topic frequently encountered involves the debate over whether or not to close laparoscopic ports, resulting in considerable variation among cases [11, 12]. Some articles even suggest a potential increase in incisional hernia incidence when ports are closed due to the complexities involved, implying heightened manipulation in the area [8, 13].

The objective of this study is to determine the incidence and characteristics of incisional hernias among patients who underwent laparoscopic bariatric surgery, as well as observe the medium and long-term progression of these hernias, along with potential discomfort and complications they may cause [14–16].

## Materials and Methods

This is a retrospective observational study with prospective recruitment of consenting patients for ultrasound evaluation in patients who underwent primary laparoscopic bariatric surgery with 12 mm trocars Endopath XCEL Bladeless Trocar (Ethicon, Johnson & Johnson, Somerville, NJ, USA) without fascial closure, including patients who agreed to participate in the study between January 2015 and July 2016 in our center, Central University Hospital of Asturias, a tertiary-level hospital which offers medical services to one million people for this surgery in a public healthcare system that guarantees almost universal coverage for all residents.

**FIGURE 1:**
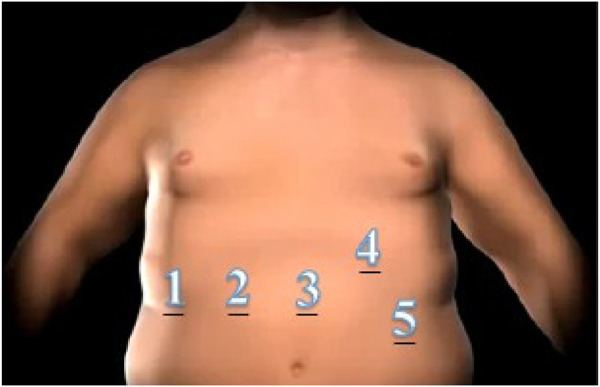
Scheme and numbering of trocars for laparoscopic approach. 1:5 mm/2–5: 12 mm ports.

The procedures performed in our hospital include laparoscopic gastric bypass and sleeve gastrectomy [17], which are conducted utilizing a configuration of 5 trocars in the same location in both surgeries, as illustrated in Figure 1. In case it is necessary to perform a minilaparotomy for the extraction of the resected stomach or introduce a circular stapler suture for the gastrojejunal anastomosis, it was closed with a continuous suture with Vicryl Sutures USP 1, Fish Hook Tapercut Needle (Ethicon, Johnson & Johnson, Somerville, NJ, USA).

We collected identified risk variables for hernia development, as well as surgical and postoperative follow-up data by reviewing electronic medical records. When offering participation in the study to patients, a telephone interview was conducted to inquire about any incidents related to surgical ports, pain, or other symptoms at that level. Physical examination was routinely performed during the first five years postoperatively during annual bariatric follow-up visits. However, we considered its diagnostic value to be limited for detecting small asymptomatic hernias, which justified the use of ultrasound [18].

Following the telephone interview, patients were scheduled for an ultrasound within a month. Data regarding post-discharge complications were collected through the electronic medical record system in our unit’s follow-up or through primary care. Surgical site infection (SSI) was defined, according to the USA Centre for Disease Control classification, as purulent material exiting the incision with a positive microbiological sample taken aseptically, with or without fever, during the first 30 postoperative days. [19]. During the interview, patients were asked to rate their pain on a scale of 1–10 [20].

**FIGURE 2:**
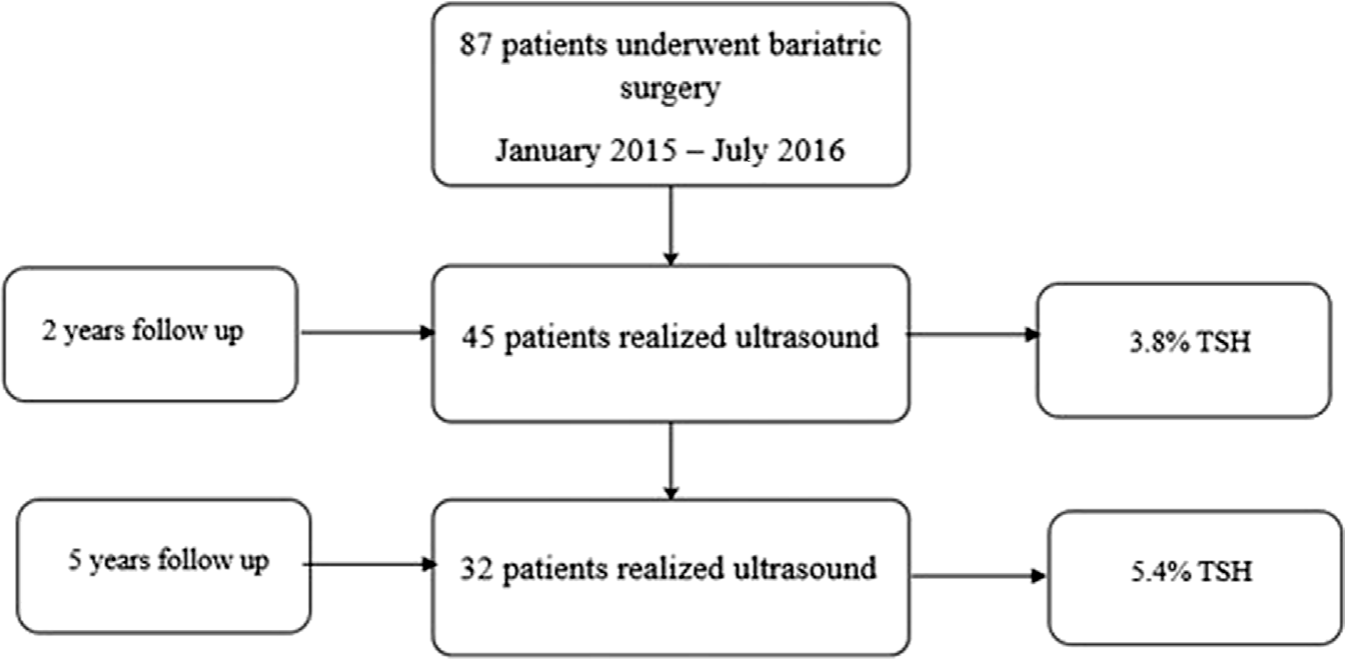
Patients undergoing study.

Furthermore, patients underwent an ultrasonography (US) study of the abdominal wall performed by an experienced radiologist in abdominal wall in the years 2018 and 2022 for the patients who consented to undergo them, using Aplio 500 or Aplio a550 (Canon) with a 10.0 MHz linear probe (Figure 2). The US diagnosis of trocar site hernia was defined as any discontinuation of the fascial layer Ultrasound examinations were performed both with and without the Valsalva maneuver and data as hernia location, size, and content were collected.

The analysis was performed using the statistical software SPSS 22. For comparisons involving categorical variables, the Chi-square test (χ2) was utilized. A significance level of p < 0.05 was considered statistically significant. An analytical study was conducted to explore associations between the various studied variables.

This study was approved by the ethics committee at “Principado de Asturias” with number 176/17. Informed consent was obtained from all participants as written informed consent.

## Results

**FIGURE 3:**
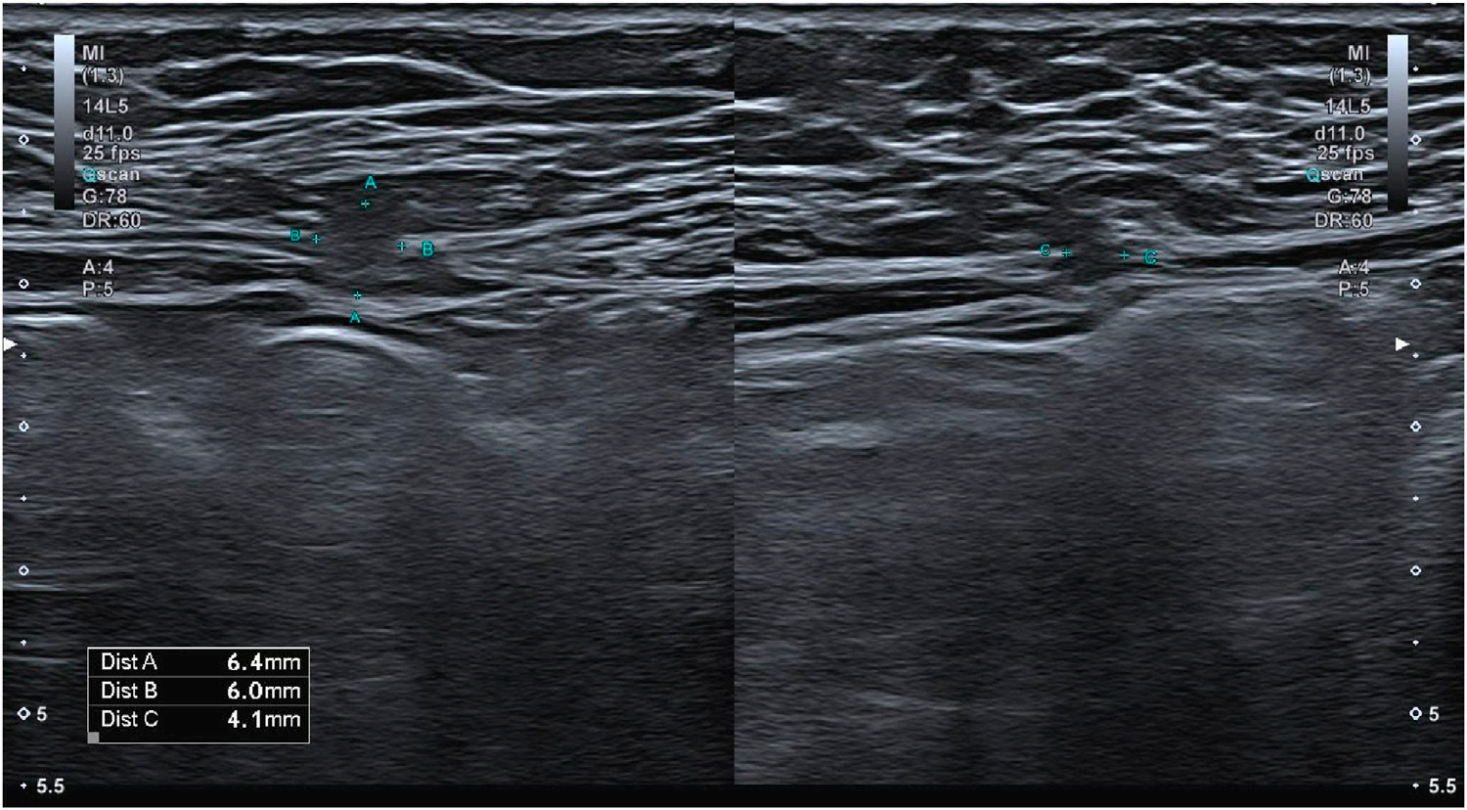
Abdominal wall visualization in ultrasound with TSH.

A total of 87 patients underwent primary bariatric surgery between January 2015 and July 2016, of whom 45 agreed to participate in the study’s first phase, with the characteristics described in Table 1. During the subsequent phase of the study, 32 patients consented to and attended their scheduled ultrasound appointments (Figure 3).

**TABLE 1:** Baseline patient and surgery characteristics^a^.

Variable (n = 45)	
Age, years, mean (SD), range	49.9 (8.6), 33–64
Gender: female/male	35 (77.8%)/10 (22.2)
BMI, kg/m^2^, mean (SD), range	45.5 (4.7), 35–55.7
Smoker	14 (31.1)
SAHS	29 (64.4)
DM	21 (46.7)
Previous abdominal surgery	14 (31.1)
Bariatric surgery Gastric bypass Sleeve gastrectomy	40 (88.9) 5 (11.1)
DOS, minutes, mean (SD), range	166.4 (52.5), 90–420
12 mm Trocars	185
5 mm Trocars	4
Minilaparotomy	36

Abbreviations: BMI body mass index; DM diabetes mellitus; SAHS sleep apnea hypopnea syndrome; DOS duration of surgery.

^a^ Values are presented as the No. (%) of patients unless otherwise specified.

The majority were women, constituting 77.8%, with a median age of 50 years and an initial mean BMI of 45.5 kg/m2. Gastric bypass was the most commonly performed surgery, accounting for 90%, with the remaining surgeries being sleeve gastrectomies.

### Mid-Term Follow-Up

The detailed results are provided in Table 2, with a median follow-up period of 26.9 months during the initial phase. In the performed ultrasound, a total of 7 hernias were detected, with a mean size of 7 mm, all of them located in trocar 3, corresponding to the optical port in the mid-epigastric area. It’s worth noticing that trocar 4 in those patients who underwent a mini-laparotomy for circular stapler gastrojejunostomy anastomosis in gastric bypass or had the sleeve gastrectomy piece removed, totaling 36 patients, was not considered susceptible of trocar hernia. However, they were still examined via ultrasound and interviews without detecting any hernia. In 2 cases, patients reported discomfort at port site 4, meanwhile another 2 patients referred pain in sites 2 and 3 without trocar site hernia. Additionally, in 4 patients trocar 1 had a diameter of 5mm, rendering it ineligible for analysis. This leaves us with a total of 185 analyzed ports, and a trocar site hernia incidence rate of 3.8%, all of them in the midline (p < 0.001) and asymptomatic.

**TABLE 2:** Postoperative and follow-up outcomes^a^.

In-hospital reintervention	0
In-hospital mortality	0
Trocar complications SSI superficial Period 1 (n = 45) Trocars: 12mm/5mm/Minilaparotomy Pain n (%), mean (SD), range 12 mm Trocar pain 12 mm Trocar hernia in mm, n (%), mean (SD), range 12 mm Trocar hernia and pain n (%) Period 2 (n = 32) Trocars: 12mm/5mm/Minilaparotomy Pain n (%), mean (SD), range 12 mm Trocar pain Trocar hernia in mm, n (%), mean (SD), range Trocar hernia and pain n (%)	11 (24.4) 185/4/36 4 (8.8), 5.5(0.5), 5–6 2/185 (1) 7/185 (3.8), 7 (4.4), 2.4–12 0 130/3/27 6 (18.7), 6.1 (1.1), 5–8 4/130 (3) 7/130 (5.4), 13.5 (6.9), 6–23 2/130 (1.5)
Follow-up in months, n (%), mean (SD), range Period 1 Period 2	45 (100), 27 (3.7), 17.3–34.2 32 (71.1), 86.1 (4.7), 78.2–94.7
EBMIL Period 2 in percentage, mean (SD), range	73.4 (23.2), 10–120

Abbreviations: EBMIL Excess Body Mass Index Loss; SSI Surgical Site Infection.

^a^ Values are presented as the No. (%) of patients unless otherwise specified.

Analysing surgical site infection (SSI), 11 patients (24.4%) showed some sign of infection, 1 in trocar 3 and mainly in trocar number 4 with 10 cases, being in 9 of these cases a mini-laparotomy for the introduction of circular stapler sutures while the remaining case was a bypass procedure with linear anastomosis using a 12 mm trocar. None of these cases did it result in a trocar hernia, representing a 25% (9 out of 36 patients) of superficial SSI in mini-laparotomies, most of them due to circular anastomosis.

In most cases, the 7 hernias visualized on ultrasound were small, with diameters of 2.4 mm, 9mm and 12 mm in two cases each and 2.6 mm in one case, with their content being fat in all cases.

In Table 3, the relationship between herniated and non-herniated patients in the first period and their respective personal backgrounds is presented.

**TABLE 3:** Herniated vs. non herniated patients in mid-term follow-up.

Variable (n = 45)	Herniated (n = 7)	Non herniated (n = 38)	p
Gender: female/male	5 female 2 male	30 female 8 male	0.6602
Smoker	3	11	0.4651
SAHS	4	25	0.6605
DM	4	17	0.5454
Previous abdominal surgery	2	12	0.8745
Bariatric surgery Gastric bypass Sleeve gastrectomy	6	34	0.7712
	1	4	

### Long-Term Follow-Up

In the ultrasound conducted during the second period, with a median follow-up duration of 85.9 months, 7 hernias were detected, with an average size of 13.5 mm. Once again, all of the hernias were located in trocar 3 and, on this occasion, 2 of the patients presented symptomatology at hernia site, but they had not sought medical attention for it.

Four of the patients with hernias had pre-existing hernias at mid-term follow-up, while 3 were newly diagnosed cases. Interestingly, out of the 3 patients who had hernias during the initial period, 2 did not have the hernias that were described in the first period, one measured 2.4 mm, the other 12 mm, and the third patient did not undergo a follow-up ultrasound. Additionally, hernias in the umbilical area were incidentally detected in 11 patients (34.3%).

No patients included in the study had undergone hernia repair prior to the ultrasound examinations, nor were emergency visits or symptoms such as incarceration reported during follow-up.

The analysis reveals a Chi-Square of 22.6 (p < 0.0001) comparing lateral ports versus epigastric port trocar site hernia in Mid-term Follow-up and 22.7 (p < 0.0001) in Long-Term Follow-Up.

## Discussion

As we mentioned previously, a trocar site hernia is defined as a defect in the abdominal wall in placements of laparoscopic ports [18]. In our study, we evaluated the incidence of trocar site hernia with an ultrasonography study performed by an expert radiologist during a 2-period follow-up in order to estimate the real incidence and evolution of this complication [20]. If we review the actually published literature, trocar site hernia incidence after a bariatric surgery is generally underestimated and has a very wide range, meanwhile with specific studies incidence rates increases from 3.2% to 6.9% [6–8].

Scozzari et al. (2014) reported a trocar site hernia incidence of 22% after Roux-en-Y gastric bypass when assessed by ultrasound, suggesting that clinical diagnosis underestimates the true incidence [21]. Similarly, Karampinis et al. (2020) in the HERBALS prospective study identified trocar site hernia incidence in 6.9% of patients using systematic ultrasound, despite port site closure. These studies highlight the underdiagnosis of trocar site hernia incidence and reinforce the value of ultrasound screening [8, 22]. In terms of trocar type, bladeless trocars are associated with lower fascial trauma, and some studies suggest that fascial closure may not be necessary for 12-mm bladeless trocars [23]. A randomized study by Ciscar et al. found no significant difference in trocar site hernia incidence between closed and non-closed ports when a prophylactic mesh was applied, questioning the automatic need for closure [8].

Comparing these data with our results, we observed that in our case represents 3.8% in the med-term follow-up and 5.4% in the long-term follow-up, reinforcing our findings based on ultrasound and clinical follow-up of a low incidence and with hardly any clinical representation, not consulting in any case for this reason and not presenting any complication related to port-site [24].

Although closure of trocars larger than 10 mm is recommended in laparoscopic surgery [25, 26], in the specific case of bariatric surgery there is not a high level of evidence about this. We could find publications with inverse relations between systematic closure of trocars and the number of hernias, in addition to an augmented risk of complications such as vascular or superficial nerve damage, as well as intestinal damage [6], facilitated by the difficulty of closure. To tackle this problem, various devices (Weck EFx^®^) and modalities have been described to facilitate closure, such as Surgicel^®^ and omental plug for prevention of trocar site hernia without facial closure [27–29].

The appearance of trocar site hernia can be both medium and long term, choosing the first moment of our study around 2 years because there is already a stabilization of weight after a process of high weight loss, and the long term [14] where patients can regain weight or we can detect hernias that may develop over the time, as well as observe the progression of those we were already aware of although in our case we achieved good results with an average of 73.4% of Excess Body Mass Index Loss [30].

There are many described risk factors in trocar site hernia appearance related to patients and surgery, as well as its complications, which we did not detect in our analysis [31]. We decided to conduct an analysis of herniated patients searching for risk factors in the first period, and due to not obtaining results, we did not pursue it in the second one. During our long-term follow-up, there was not any significative complication related to trocar site hernia. Most hernias increased in size over the years, as anticipated, although they persist at a small size with minimal symptomatology.

The location of the hernias was in all cases in the midline (trocar 3), which makes them have a very marked statistical representation with hardly any clinical representation, probably due to their location in the upper abdomen and anatomically close to the round ligament of the liver, reducing the possibilities of incarceration and being occupied by preperitoneal fat from the beginning.

We did not find any hernias in minilaparotomies incisions, even with a 25% (9 out of 36 patients) incidence of infection, most of them in bypass surgery with circular stapler anastomosis. These data are one of the reasons why we currently perform linear anastomosis and we encourage other bariatric surgery groups to abandon circular stapler anastomosis.

Ultrasound is the gold standard in the detection of trocar site hernia [24, 25]. The reason why there are two patients in which hernia was detected in the first period and not in the second one may be justified in one case by the size of 2.4mm, but in the second case of 12 mm we can speculate about the healing process in the long term or technical or transcription error, without actually knowing the answer. In the long term, it is not exempt from new hernias similar to the ones detected, which is logical to expect may be larger in size. Interestingly, there is a high number of long-term hernias in the umbilical region that are diagnosed incidentally, probably related to some of the patients undergoing abdominoplasty after their bariatric surgery. This data is beyond the scope of this work and that may be susceptible of further studies in the future.

Although pain is a primary symptom of abdominal wall hernias, and patients reported experiencing pain greater than 5 on a 10-point scale during the telephone interview, none sought medical consultation for this symptom [32]. This occurred despite the fact that our surgical follow-up is conducted annually for the first 5 years postoperatively, and healthcare access in our country is both universal and free of charge. That results makes us think about the augmented pain tolerance of these patients, who come from a low quality of life before surgery [17], as well as the subjective nature of the symptoms, which in the first period were not related to trocar site hernia and in the second period of follow-up the percentage of this relation was low.

In one hand, the main strengths of our study were that the same port situation were maintained for every surgery performed, as well as the same surgical materials, the follow up was monitored by a single researcher and the support of an US provided more accurate diagnosis of trocar site hernia by the same team of radiologists.

On the other hand, the limitations of our study include the lack of patient randomization, the inclusion solely of patients who voluntarily agreed to participate, the inability to track all patients from the initial period, a small study population, which are potential source of bias, as well as it was conducted at a single institution and may not be a representative sample.

### Conclusion

The incidence of trocar hernia in the medium and long term after laparoscopic bariatric surgery with 12 mm bladeless trocars without fascial closure in our community is low, all of them being in the midline and asymptomatic. These results lead us to abstaining from closing the transmuscular accesses, and they also prompt us to contemplate a repositioning or to deliberate on the systematic closure of the epigastric trocar, particularly the midline optical port.

## Data Availability

The original contributions presented in the study are included in the article/supplementary material, further inquiries can be directed to the corresponding author.
